# Phospholipid Membrane-Mediated Hemozoin Formation: The Effects of Physical Properties and Evidence of Membrane Surrounding Hemozoin

**DOI:** 10.1371/journal.pone.0070025

**Published:** 2013-07-23

**Authors:** Nguyen Tien Huy, Yusuke Shima, Atsushi Maeda, Tran Thanh Men, Kenji Hirayama, Ai Hirase, Atsuo Miyazawa, Kaeko Kamei

**Affiliations:** 1 Department of Applied Biology, Kyoto Institute of Technology, Sakyo-ku, Japan; 2 Department of Clinical Product Development, Institute of Tropical Medicine (NEKKEN), Nagasaki University, Nagasaki, Japan; 3 Program for Nurturing Global Leaders in Tropical and Emerging Communicable Diseases, Nagasaki University, Nagasaki, Japan; 4 Department of Biomolecular Engineering, Kyoto Institute of Technology, Sakyo-ku, Japan; 5 Department of Immunogenetics, Institute of Tropical Medicine (NEKKEN), Nagasaki University, Nagasaki, Japan; 6 Bio-multisome Research Team, RIKEN SPring8 Center, Harima Institute, Sayo, Japan; Liverpool School of Tropical Medicine, United Kingdom

## Abstract

Phospholipid membranes are thought to be one of the main inducers of hemozoin formation in *Plasmodia* and other blood-feeding parasites. The “membrane surrounding hemozoin” has been observed in infected cells but has not been observed in *in vitro* experiments. This study focused on observing the association of phospholipid membranes and synthetic β-hematin, which is chemically identical to hemozoin, and on a further exploration into the mechanism of phospholipid membrane-induced β-hematin formation. Our results showed that β-hematin formation was induced by phospholipids in the fluid phase but not in the gel phase. The ability of phospholipids to induce β-hematin formation was inversely correlated with gel-to-liquid phase transition temperatures, suggesting an essential insertion of heme into the hydrocarbon chains of the phospholipid membrane to form β-hematin. For this study, a cryogenic transmission electron microscope was used to achieve the first direct observation of the formation of a monolayer of phospholipid membrane surrounding β-hematin.

## Background

Malaria is one of the most common diseases in tropical countries. Each year, there are an estimated 225 million new malaria infections and almost a million deaths due to malaria world-wide [1]. Spreading resistance to current quinoline antimalarials and artemisinine has made malaria a major global problem [2]. Since a vaccine for malaria is not available, it is essential to study the molecular, biochemical, and immunological aspects of malarial parasites to develop vaccines and new antimalarial drugs.

Host protein digestion has two aspects: to obtain amino acids and to regulate osmotic pressure. This hemoglobin digestion takes place in the parasites’ food vacuoles and is carried out by multiple proteases including four aspartic versions [3]: three cysteine proteases [4] and a zinc metalloprotease (falsilysin) [5]. These proteases digest hemoglobin into small fragments consisting of about 20 different amino acids and free ferrous protoporphyrin IX (Fe(II)PPIX), which is rapidly oxidized to Fe(III)PPIX (heme). Heme is the deep red, oxygen-carrying, non-protein, ferrous component of hemoglobin in which the iron is Fe(II) (ferrous iron) and also called reduced hematin. Iron(III), ferriprotoporphyrin IX, (Fe(III)PPIX) is known to be present in solution as hematin (H2O/OH-Fe(III)PPIX). The free heme is oxidatively active and toxic to both the host cell and the malarial parasite, however free heme is rapidly oxidized to hematin and sequestered into hemozoin (malarial pigment). Due to the absence of heme oxygenase, the parasite is unable to cleave heme into an open-chain tetrapyrrole, which is necessary for cellular excretion [6]. To protect itself, the malarial parasite detoxifies free heme *via* neutralization with a histidine-rich protein 2 [7], [8], degradation with reduced glutathione [9], [10], [11], or crystallization into hemozoin which is a water-insoluble malarial pigment that is not lethal to biological cells [7], [12]. However, at least 95% of free heme in *P. falciparum* is reportedly converted to hemozoin [13], [14]. Hemozoin is known to be structurally and chemically identical to *in vitro* synthetic β-hematin (BH), which is a crystal of the heme (Fe(III)PPIX) dimer of the hematin Fe (III) PPIX dimer [15], [16], [17]. It has been used for parasite concentration and detection [18], [19], [20], [21], [22]. It is also suggested that the blocking of BH formation is an ideal target for antimalarial screening [23], [24], [25], [26]; thus, it is important to understand the mechanism of BH formation.

Several factors such as histidine-rich protein [7], [8], elevated temperature [27], lipids [28], [29], pre-formed BH [25], alcohols [30], detergent [31], and malarial heme detoxification protein [32] are reportedly responsible for heme crystallization. Among these factors, lipid droplets and phospholipid membranes are proposed as the main inducers of hemozoin formation in *Plasmodia* and other blood-feeding parasites including *Schistosoma* and *Rhodnius* [33], [34], [35], [36], [37], [38], [39], [40], [41]. The mechanism of BH formation induced by neutral lipid droplets both *in vivo* and *in vitro* has been well documented [34], [35], [36], [37], [38]. The “membrane surrounding hemozoin” has been found in the *in vivo* ultrastructure [39], [41], [42], but it has not been observed in *in vitro* experiments. In the present study, we aimed to observe the association of phospholipid membranes and BH crystals, and to further explore the mechanism of phospholipid membrane-induced BH formation.

## Materials and Methods

### Materials

Hemin chloride (heme) was purchased from Sigma. L-α-Phosphatidylcholine dilauroyl (dilauroyl-PC), L-α-phosphatidylcholine dimyristoyl (dimyristoyl-PC), L-α-phosphatidylcholine dipalmitoyl (dipalmitoyl-PC), L-α-phosphatidylcholine distearoyl (distearoyl-PC), L-α-phosphatidylcholine dioleoyl (dioleoyl-PC), L-α-phosphatidylserine dipalmitoyl (dipalmitoyl-PS), L-α-phosphatidylethanolamine dimyristoyl (dimyristoyl-PE), L-α-phosphatidylethanolamine dipalmitoyl (dipalmitoyl-PE), dimethyl sulfoxide, and chloroform were provided by Wako Pure Chemicals (Osaka, Japan). The remaining reagents were also acquired from Wako Pure Chemicals.

### Preparation of Lipid Vesicles

Phospholipids were dissolved in 1 ml chloroform at a concentration of 2 mM. Then they were sprayed and dried on the walls of 1.5 ml micro-tubes under a nitrogen gas flush to create a thin layer, which was then suspended in 1 ml of distilled water. The lipid suspension (2 mM) was sonicated for 10 s and used for BH formation assay, as previously described [43], and for cryo-TEM observation.

### Assay of BH Formation Initiated by Phospholipids

Stock heme solution (10 mM) was prepared using hemin chloride in dimethyl sulfoxide as described previously [10]. Heme (100 µM) was incubated with various concentrations of phospholipids in 1 ml of 50 mM acetate buffer at pH 4.8. For quantification of BH, after incubating at 37°C for 16 h, the sample was centrifuged for 5 min at 7,000×*g*, and the supernatant was discarded. The obtained BH was purified and quantified as previously described [44], [45]. Values obtained from triplicate assays were plotted, and the EC_20_ values (M), along with the concentration of lipids required for crystallizing 20% of the heme, were calculated graphically. The characteristics of BH were confirmed by infrared spectroscopy with expected infrared spectra peaks at 1210 and 1664 cm^−1^, confirming the presence of BH.

For cryo-TEM observation, the heme (100 µM) in the acetate buffer (0.5 M) and that in the phospholipid suspension (0.5 mM) was mixed and incubated at 37°C for 6 h. Then, 2.5 µl of the BH precipitate that had formed in the bottom of the tube was used.

### Physical and Chemical Properties of Phospholipids

The gel-to-fluid phase of the transition temperature of phospholipids (*T*_m_) was derived from a procedure established by Cevc *et al.* [46]. The molecular weight was recorded from the supplier (Wako Pure Chemicals). Other physical properties of the phospholipids, including total net charge, number of anion charge, octanol-water partition coefficient (log*P*), distribution coefficient (log*D* at pH 5.5), hydrogen bond acceptors, hydrogen bond donors, freely rotating bonds, polar surface area, and polarizability were retrieved from ChemSpider (www.chemspider.com), as predicted by Advanced Chemistry Development (ACD/Laboratories) software.

### Cryo-transmission Electron Microscopy (cryo-TEM)

The analysis was performed as described previously [47], [48]. Briefly, samples (2.5 µL) were applied to glow-discharged microgrids supported by 5-nm-thick carbon films (JEOL, Tokyo, Japan). After removing excess samples with pre-water-soaked filter paper, the samples were quickly frozen by liquid ethane cooled by liquid Nitrogen (EM CPC, LEICA Microsystems, Vienna). The grid was then transferred into a JEOL cryo-electron microscope (JEM3000SFF) and kept at 4.2 K and observed at 300 kV.

### Statistical Analysis

Data analysis was performed using the SPSS Version 14. The Pearson correlation was analyzed to evaluate the relationship between the abilities of phospholipids to induce BH formation and their physical properties. Differences in BH formation induced by lipids were analyzed for statistical significance using the nonparametric Mann–Whitney U test. Values were considered significant at p<0.05.

## Results

### BH Formation Induced by Phospholipids

**Figure 1:**
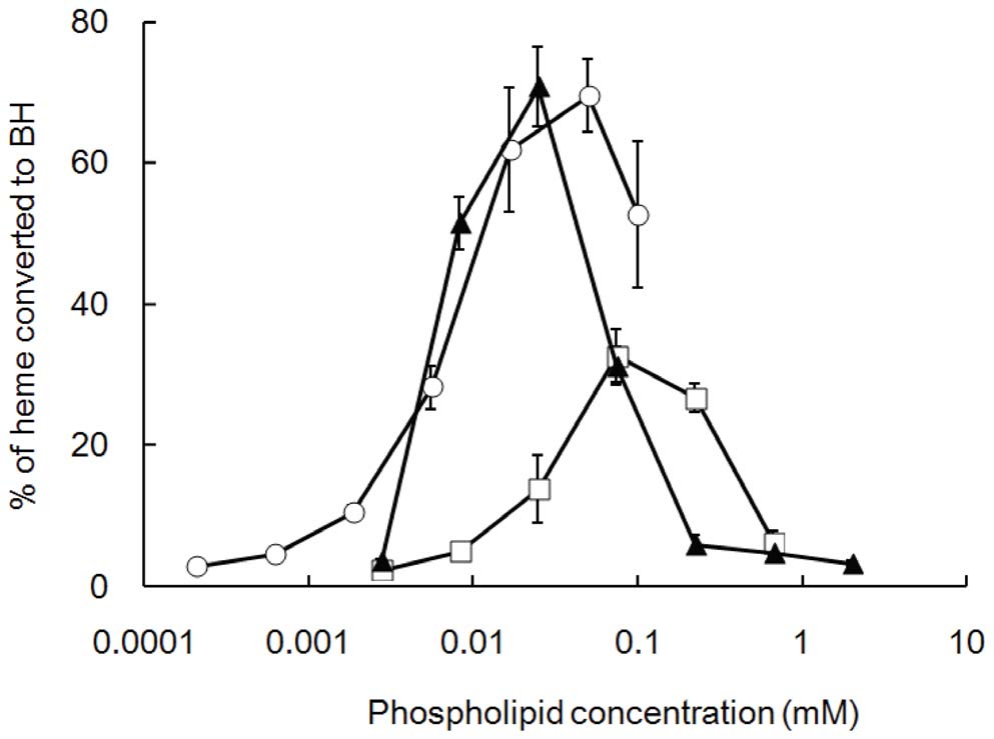
BH formation induced by various concentrations of phospholipids at 37°C. The ordinate shows the amount of BH produced by incubation with various concentrations of phospholipids for 16 h, as expressed as the percentage of heme converted to BH. Values represent the mean ± SD (n  = 3). The results were reproducible. Circle; dioleoyl-PC, triangle; dilauroyl-PC, square; dimyristoyl-PC.

The abilities of various phospholipids to induce BH formation *in vitro* at 37°C are shown in Fig. 1. BH formation was induced by phospholipids in a biphasic dose-dependent manner. Two phosphatidylcholines, dilauroyl-PC and dioleoyl-PC, were catalyzed by BH formation at low molar concentrations and converted a maximum of 70–80% of the heme into BH, indicating a relatively high efficiency. Dimyristoyl-PC converted a maximum of 30–40% of the heme into BH at a slightly higher concentration of the inducer. The concentration that is required to convert 20% of heme into BH (EC_20_ values) for these phospholipids varied from 5 to 12 µM (Table 1). We also observed that the maximal yield of BH in the presence of lipids was negatively correlated with the EC_20_ values (Fig. 1). In contrast, dipalmitoyl-PC, distearoyl-PC, dimyristoyl-PE, dipalmitoyl-PE, and dipalmitoyl-PS could not induce BH formation under our experimental conditions.

**Table 1:** BH-forming ability and the physical properties of phospholipids.

Phospholipids	Acyl groups	*T*_m_ (°C)^a^	MW^b^	Total net charge	No. of anion charge	log*P* c	log*D* c (pH 5.5)	Hydrogen bond acceptors^c^	Hydrogen bonddonors^c^	Freely rotating bonds^c^	Polar surface area (angstrom^2^)^c^	Polarizability^c^ (×10^−24^)	BH induction (EC_20_ values, mM)^d^
**dilauroyl-PC**	12∶0	0	621.8	0	1	6.62	7.23	9	1	32	118.17	1	6
**dimyristoyl-PC**	14∶0	23	677.9	0	1	8.05	8.65	9	1	36	121	1	12
**dipalmitoyl-PC**	16∶0	42	734.0	0	1	10.09	10.69	9	1	40	121	1	ND
**distearoyl-PC**	18∶0	55	790.1	0	1	12.12	12.73	9	1	44	121	1	ND
**dioleoyl-PC**	18∶1	−22	788.1	0	1	11.96	12.57	9	1	42	121	1	5
**dimyristoyl-PE**	14∶0	48	635.8	0	1	11.00	8.51	9	3	36	144.190	68.93	ND
**dipalmitoyl-PE**	16∶0	63	691.9	0	1	13.70	11.20	9	3	40	144.19	76.276	ND
**dipalmitoyl-PS**	16∶0	51	735.9	−1	2	13.04	9.55	11	4	41	181.49	78.717	ND

a T*_m_*: Phase transition temperature was derived from Cevc et al (1993) in Phospholipids Handbook (Cevc, G., ed.), pp. 939–956, Marcel Dekker, New York.

b given by supplier.

c Values were retrieved from ChemSpider (www.chemspider.com), predicted by Advanced Chemistry Development (ACD/Laboratories) software.

d performed by this study.

### Effects of Physical and Chemical Properties of Phospholipids on BH Formation

To further understand the mechanism of phospholipid-mediated BH formation, Pearson correlation analysis between the abilities of phospholipids to induce BH and their physical properties was performed. All phospholipids were tested at the same temperature (37°C). Our results showed that the ability of phospholipids to induce BH formation was inversely correlated with the gel-to-fluid phase transition temperature (*T*_m_) of phospholipids (Table 1, p>0.05). Moreover, only phospholipids with a *T*_m_ that was lower than the experimental temperature (37°C) could induce BH formation. Phospholipids such as dioleoyl-PC with a lower *T*_m_ were more effective in inducing BH formation than phospholipids such as dilauroyl-PC, which had a higher *T*_m_.

In contrast, other physical properties of phospholipids were not significantly associated with the ability of phospholipids to induce BH formation: molecular weight, total net charge, number of anion charge, octanol-water partition coefficient (log*P*), distribution coefficient at pH 5.5, hydrogen bond acceptors, hydrogen bond donors, freely rotating bonds, polar surface area, and polarizability (Table 1, p>0.05).

### Effect of Reaction Temperature on Phospholipid-mediated BH Formation

**Figure 2:**
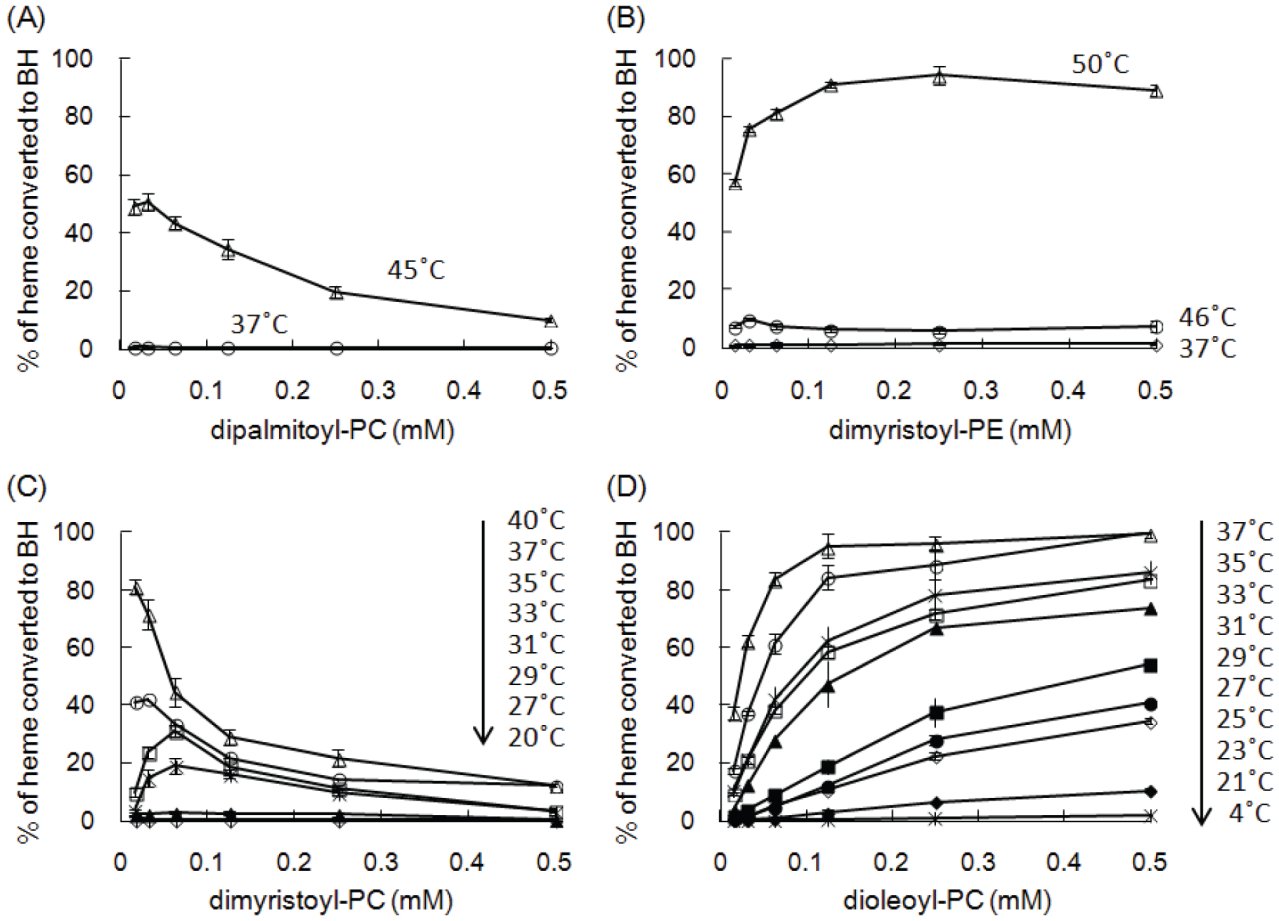
Effect of reaction temperature on phospholipid-mediated BH formation. Dipalmitoyl-PC (A), dimyristoyl-PE (B), dimyristoyl-PC (C), and dioleoyl-PC (D) were used to induce BH formation at various temperatures, and the reaction was dependent on the phase transition temperature of phospholipids. *T*_m_ values are 42°C for dipalmitoyl-PC (A), 48°C for dimyristoyl-PE (B), 23°C for dimyristoyl-PC (C), and −22°C for dioleoyl-PC (D). It is noted that BH was not formed at 50°C in the absence of phospholipids. Values represent the mean ± SD (n  = 3).

To further clarify the relationship between the phase transition temperature of phospholipids and their abilities to induce BH, we performed a BH formation assay at temperatures ranging from below to above the *T*_m_ of a particular phospholipid. Since BH formation spontaneously occurs with no inducer at high temperature [49], distearoyl-PC, dipalmitoyl-PE, and dipalmitoyl-PS could not be tested in this experiment because of their high *T*_m_ values. We further confirmed that spontaneous BH formation was not observed at 50°C in the absence of phospholipids. The results shown in Fig. 2 indicate that all tested phospholipids could induce BH formation at a temperature higher than the *T*_m_ of a particular phospholipid in the liquid phase. On the other hand, no BH was formed with a reaction temperature below the *T*_m_ of a particular phospholipid in the solid phase. Furthermore, the maximal yield of BH was positively correlated with the reaction temperature. These results suggested that the ability of phospholipid vesicles to induce BH formation was correlated with their membrane fluidity.

### Cryo-TEM Analysis

**Figure 3:**
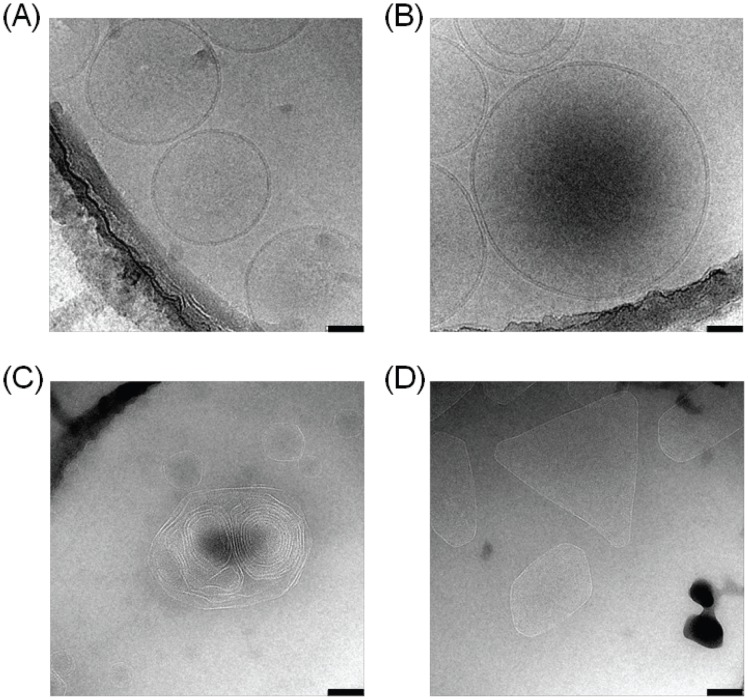
Cryo-TEM images of lipid liposomes prepared in water at 37°C. Liposome of dioleoyl-PC (A and B) or dipalmitoyl-PC (C and D) prepared in water by ultrasonication was observed by cryo-TEM. Scale bars are 50 nm in Figs. A and B and 100 nm in Figs. C and D.

To examine whether the BH-inducing ability of phospholipid vesicles was related to structure, observations by cryo-TEM were performed. The dioleoyl-PC liposome was mostly observed as spherical vesicles with a bilayer membrane (Fig. 3A), while a few multilamellar liposomes were also observed by cryo-TEM (Fig. 3B). However, dipalmitoyl-PC liposome was also observed, but the large liposome (diameter >100 nm) was irregular and angular, probably indicating less fluidity compared with dioleoyl-PC liposome (Figs. 3C and D).

**Figure 4:**
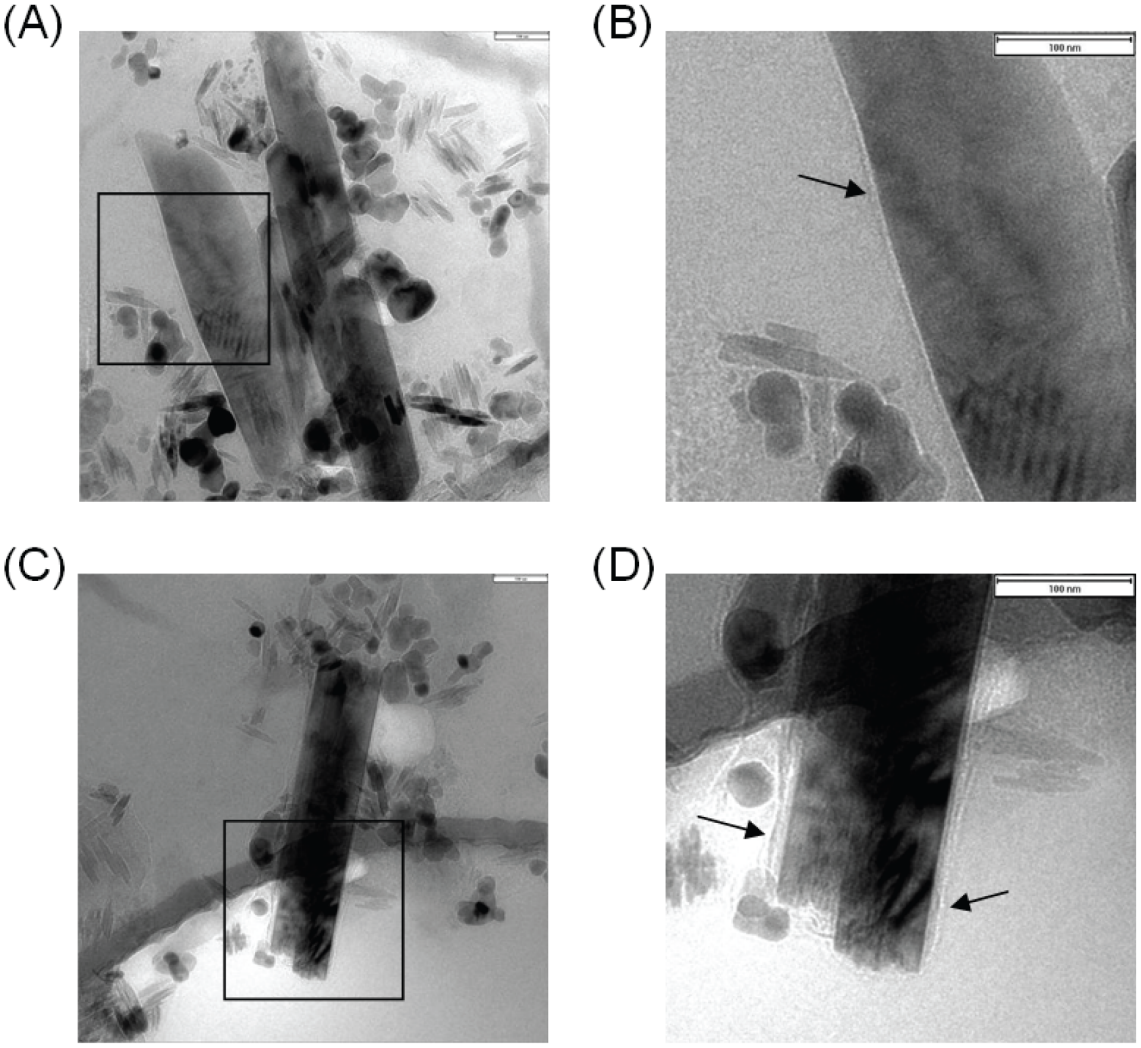
Cryo-TEM images of BH catalyzed by dioleoyl-PC liposome. Heme (100 µM) was incubated with dioleoyl-PC liposome in 1 ml of acetate buffer at pH 4.8. After incubation for 16 h at 37°C, the samples were observed by cryo-TEM as described in the Methods section. Figs. B and D are magnified images of the squared area in Figs. A and C, respectively. Black arrows in Figs. B and D indicate the monolayers of the phospholipids. Scale bars are 100 nm.

The formation of BH catalyzed by dioleoyl-PC liposome was also observed using cryo-TEM. The cryo-TEM images of BH showed morphologies that were almost identical (Fig. 4). The BH appeared as variable-sized crystals with long thin shapes, smooth surfaces and tapered ends. These morphological characteristics were similar to those seen in previous reports of BH both *in vivo* and *in vitro* [50], [51], which further confirmed the BH formation. The dioleoyl-PC liposome was not observed around the BH, but a monolayer membrane-like structure that surrounded the BH was observed, and is indicated by arrows in Figs. 4B and 4D. With longer irradiation from an electron beam, this membrane-like structure was burned and developed a white color, as observed under cryo-TEM. Given the fact that only the dioleoyl-PC in the mixture had the potential to form a monolayer structure, this observation strongly suggests that the membrane-like structure was formed by a phospholipid.

## Discussion

Numerous studies have suggested that neutral lipid droplets are the main templates for hemozoin formation [34], [35], [36], [37], [38]. For many years, phospholipid membranes have also been proposed as the site of hemozoin formation in malaria [39], [40]. Recently, Kapishnikov *et al.* used cryogenic soft X-ray tomography to demonstrate the hemozoin formation templates on the inner layer of a digestive vacuole [41]. However, very few studies have investigated BH formation induced by phospholipid membranes in an *in vitro* experiment [40], [52]. As far as could be ascertained, the present study is the first to observe a membrane surrounding BH that was induced by phospholipid vesicles (Fig. 4), which was a structure that was similar to that observed *in vivo* by Hempelmann *et al.* [39]. The advantage of cryo-TEM was that it allowed the direct observation of phospholipid membranes and BH formation with no fixation or dehydration of the sample on the grid. In addition, cryo-TEM was suitable for the detection of phospholipid membranes because the phosphorus groups have a low permeability to the electron beam. Moreover, the association of polar lipids with malarial hemozoin observed by the thin layer chromatography also supports our results [53].

The differences in morphology between dioleoyl-PC and dipalmitoyl-PC vesicles, as observed by cryo-TEM, suggested that a smooth regular shape of vesicles is required for the site of a BH template. The differences in morphology between dioleoyl-PC and dipalmitoyl-PC vesicles were probably due to their different physical properties, in particular the different states – fluid or gel – caused by the differences in *T*_m_ values. Moreover, since the hydrophobic interactions between their inducers and heme have been proposed as an important force in the creation of a precursor heme dimer [38], [45], [54], [55], it was interesting to explore the correlation between the physical properties of phospholipids and their ability to induce BH. Our results demonstrated that phospholipids could induce BH formation only in the fluid phase, and could not do so in the gel phase. Recently, Hoang et al. showed that a blending of five neutral lipids lower the melting temperature of lipid droplets compared to the homogeneous samples and the blending of five neutral lipids produced more hemozoin compared to that of homogeneous lipids, further supporting the role of lipid fluidity in the hemozoin formation.

Evidence showed that an increase in membrane fluidity results in the membrane insertion of heme [56], which is positively correlated with an increase in the BH induction of phospholipids (Fig. 2). These observations suggest that the mechanism of BH formation involves the acyl chains of the phospholipid membranes. Furthermore, free heme can be quickly and easily inserted into phospholipid vesicles as monomeric heme at a ratio of 1 heme per 4–5 phospholipid molecules in the fluid phase [57], [58]. Taken together, these observations suggest that the free heme inserts its vinyl groups deeply into the hydrophobic acyl chains of phospholipids while the charged propionate groups are exposed to the aqueous solution. The hydrophobic environment of acyl chains helps to form monomeric heme, which favors the formation of a BH dimer. However, other mechanism cannot be excluded, and further studies are needed to clarify this issue.

A biphasic dose-dependent manner of BH formation induced by phospholipids was also observed in the assay induced by a detergent [31], probably due to the over dilution of the heme molecules in the high number of vesicles. Further studies are required to clarify this mechanism.

### Conclusions

Our results showed that the abilities of phospholipids to induce BH is inversely correlated with the phase transition temperatures, suggesting a required insertion of heme into the hydrophobic acyl chains of a phospholipid membrane in order to form BH. Finally, a monolayer of membrane surrounding BH was observed using cryo-TEM.
